# Allelopathy as a potential strategy to improve microalgae cultivation

**DOI:** 10.1186/1754-6834-6-152

**Published:** 2013-10-21

**Authors:** Leonardo Brantes Bacellar Mendes, Alane Beatriz Vermelho

**Affiliations:** 1 Leopoldo Américo Miguez de Mello Research and Development Center, CENPES, PETROBRAS, Rio de Janeiro, Brazil; 2 Department of General Microbiology, Institute of Microbiology Paulo de Góes, Federal University of Rio de Janeiro, Rio de Janeiro, Brazil; 3 BIOTECHNOLOGY CENTER – BIOINOVAR: Bioenergy, Biocatalysis and Bioproducts Unit, Federal University of Rio de Janeiro, Rio de Janeiro, Brazil

**Keywords:** Microalgae, Allelopathy, Biodiesel, Contamination, Allelochemicals, Cultivation

## Abstract

One of the main obstacles for continuous productivity in microalgae cultivation is the presence of biological contaminants capable of eliminating large numbers of cells in a matter of days or even hours. However, a number of strategies are being used to combat and prevent contamination in microalgae cultivation. These strategies include the use of extreme conditions in the culture media such as high salinity and high pH to create an unfavorable environment for the competitive organisms or predators of the microalgae. Numerous studies have explored the potential of naturally occurring bioactive secondary metabolites, which are natural products from plants and microorganisms, as a source of such compounds. Some of these compounds are herbicides, and marine and freshwater microalgae are a source of these compounds. Microalgae produce a remarkable diversity of biologically active metabolites. Results based on the allelopathic potential of algae have only been described for laboratory-scale production and not for algae cultivation on a pilot scale. The adoption of allelopathy on microalgal strains is an unexplored field and may be a novel solution to improve algae production. Here we present information showing the diversity of allelochemicals from microalgae and the use of an allelopathic approach to control microalgae cultivation on a pilot scale based on R&D activities being carried out in Brazil for biodiesel production.

## Introduction

Currently, sustainability is a cornerstone in the management of natural resources. The availability of these resources, the environmental impacts, and the socio-economic relationship resulting from their use in industrial processes need to be considered. The scarcity of fossil fuels has promoted a search for renewable and alternative low-cost energy sources [1]. Microalgae are organisms that have rapid growth rates and the ability to accumulate and synthesize 20 to 50% of their dry weight in neutral lipids, which are stored in the cytoplasm in the form of lipid bodies (lipid droplets) or lipid inclusions [2]. These features justify the use of microalgae for biodiesel production through the transesterification of lipids in a non-toxic and biodegradable process that may be chemical or enzymatic [3]. The use of microalgae might contribute to reduce the carbon dioxide emissions from fossil fuels, thus reducing the impact of global warming. Microalgae exhibit increased photosynthetic efficiency and higher growth rates than higher-order plants: they are capable of completing a growth cycle within a few days [4]. Oils from different origins have been used for producing biodiesel. Nevertheless, the cultivation of plants for oil extraction faces competition from the food and animal feed industries for the use and occupation of land [5]. The use of microalgae for biodiesel production has long been recognized and its potential has been reported in many recent studies and besides this, microalgae have multiple industrial applications [4,6-8]. However, current microalgal mass culture technologies have failed to produce bulk volumes of microalgal biomass at low costs due to contamination by biological pollutants [9]. The presence of contaminants is a major obstacle in biofuel production on an industrial level, especially in open-air ponds. The use of biocides and antimicrobial substances, with a selective spectrum for algae, protozoa, fungi, viruses, grazers and bacterial contaminants, is required to maintain continuous production and ensure quality control of these cultures. Allelopathy is a new approach and a promising strategy to achieve the goal of producing bulk volumes of microalgae. In this review we present information on the biotic and abiotic factors required for the cultivation of microalgae and discuss the current potential methods of contamination control, with an emphasis on the possible use of allelopathy and allelochemicals [10,11].

### Cultivation of microalgae

Microalgae constitute unicellular or multicellular organisms belonging to a group of prokaryotic or eukaryotic photosynthetic microorganisms that include cyanobacteria, blue-green alga (gram-negative bacteria), and green microalgae and diatoms [12]. Prokaryotic microalgae (cyanobacteria) lack membrane-bound organelles (plastids, mitochondria, nuclei, Golgi bodies, and flagella) whereas in eukaryotic microalgae, which include many different types of common algae, these organelles control cellular functions. It is estimated that there are more than 50,000 species of microalgae worldwide, among which about 30,000 have been studied [5]. Algae can either be autotrophic or heterotrophic. While autotrophic algae need inorganic compounds such as CO_2_, salts, and light as energy sources for growth, heterotrophic algae, which are non-photosynthetic, require external sources of organic compounds and nutrients for their energy supply. A third type of algae is mixotrophic as they have the ability to acquire exogenous organic nutrients or perform photosynthesis, depending on the environmental conditions [1].

The basic needs for the cultivation of microalgae are light, CO_2_, minerals, and water, but different factors may be involved in their growth depending on the species or strain. Microalgae may be cultivated in open systems or photobioreactors. Aside from carbon, in the form of carbon dioxide, microalgae require nitrogen, which is associated with their primary metabolism. Fast-growing species of microalgae prefer using ammonia instead of nitrate. Partial depletion of nitrogen is associated to lower growth rates and greater lipid production in microalgae. Lipids are synthesized as a reserve for situations of nutritional stress. Phosphorus is the third most important nutrient for algae and should be added to the medium in the form of phosphates. In addition to these components, trace metals such as Mg, Ca, Mn, Zn, Cu, and Mb, and vitamins are added to the culture medium for higher productivity [4,5,13]. Higher temperatures accelerate microalgae metabolism while lower temperatures lead to inhibition. The optimum temperature for many species of microalgae is in the 15–26°C range. Most microalgae prefer a neutral pH, but *Spirulina platensis*, among other species, prefers a pH of 9.0 while others such as *Chlorococcum littorale* grow best at pH 4.0. Due to their fast growth rate, algae very quickly attain high densities. Thus, for light to reach these dense growths, stirring apparatuses to agitate the algae and cause water circulation are necessary [14].

### Open and closed cultivation systems

**Figure 1:**
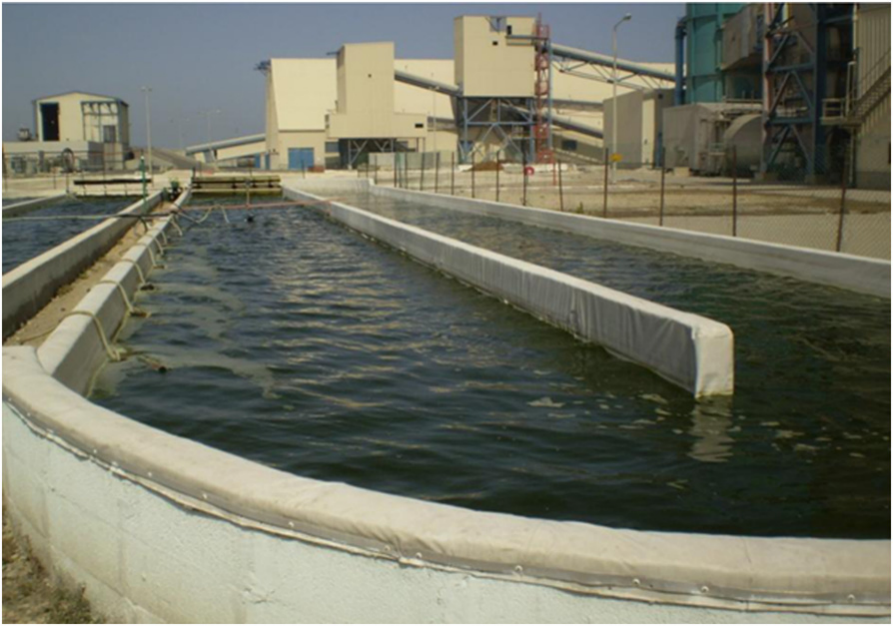
**Cultivation of *Nannochloropsis *** **sp. microalgae using seawater in open systems at the Seambiotic company, Israel, 2009.**

A major bottleneck for the cultivation of microalgae for biofuel production is to achieve industrial-level production. This is necessary to make the process economically feasible and to ensure an uninterrupted supply for consumer markets [1]. Currently, most of the worldwide commercial production of microalgae is carried out in open ponds [15] (Figure 1).

Open systems, also known as “open ponds”, have been much improved and have significant advantages over closed systems [1]. Additionally, the open systems are economically advantageous, but have problems of contamination from protozoa, viruses, other species of algae, fungi, and airborne micro-organisms, besides not allowing the control of the water temperature and light. The closed systems, called photobioreactors, which after numerous advances in materials engineering and applied biotechnology of the latest generation, are currently employed to produce fine chemicals (e.g., Astaxanthin), with a high added value (reaching up to U$ 6,000.00/kg) for commercialization. The main advantage of the photobioreactors is that the physical and nutritional parameters can be controlled [6,16]. Table 1 shows the advantages and disadvantages of each system.

**Table 1:** Comparison of cost and efficiency of open ponds and bioreactors for microalgae cultivation

***Factors***	***Open ponds***	***Bioreactors***
Equipment	Low cost	High cost
Investment	Low	High
Operating Costs	Low	High
Maintenance and control of temperature	No cost but difficult	Easy but costly
Scaling up	Easy	Difficult
Agitation of large volumes of algae	Difficult, low uniformity, and low cost	Easy, high uniformity, and high cost
Evaporation of culture medium	High	Low
Risk of contamination	High	Reduced
Control of contamination	Difficult	Easy
Control of species	Difficult	Easy
Microalgae stress	Low	High
Maintenance and cleaning of the system	Easy	Difficult
Productivity	Low	High

### Biological contamination

To be successful, engineering techniques applied to the construction and development of photobioreactors need to rely on the knowledge we have concerning the behavior and microbiology of that particular strain of microalgae, including its genetic makeup and metabolic pathways. However, the cultivation of microalgae in open systems is only possible with the use of strategies that prevent biological contamination, predators/grazers, and parasites of various origins. We still know very little about the physiology and behavior of microalgae, and this field of biotechnology is still in its early stages. Additionally, the potential of culture contamination is a constant threat. Fungi, protozoa, and bacteria can cause contamination or other species of microalgae can enter the system and compete with the main microalgae [17]. Contamination can come from the air, dust particles, leaves, and other materials brought by the wind, as well as insects. These last are a major source of contamination.

Contaminants may not directly affect the production of algae but they may affect the pH, which may lead to cultivation limitations. Some examples of biological contaminants were recently reported by JR Benemann at an international meeting held in Denver/USA (Algae Biomass Summit, 2012). He suggested that these contaminants were key issues to be studied and that better solutions to control them should be investigated. Contaminants found in open ponds include amoebae, ciliates, flagellates, rhizopods (protozoa), and other algae such as *Oocystis* sp. and *Dunaliella viridis*, Artemia and Parartemia (zooplankton), as well as viruses. Viruses are present in aquatic environments and can be found in association with both eukaryotic algae and cyanobacteria. Viral infection can reduce an algal population within a few days [9]. Insects of the order Diptera (flies and mosquitoes) and aquatic insects of the order Hemiptera (beetles) may also be present. Protozoa are predators rather than contaminants and some of them such as *Paramecium bursaria* contain symbiotic cells of *Chlorella* sp. [18,19]. Cultivation in open ponds is susceptible to grazing by zooplankton, which can reduce algal concentrations and production to low levels within just a few days [9,20]. Among these zooplankton, ciliate [21], rotifer [22] cladoceran, and copepod [23] are the most common predatory species in microalgae cultivation [9]. The two types of feeding mechanism used by zooplankton have been observed in copepods: a passive (microalgae flowed into the copepod) and an active mechanism (copepod maxillae movement to bring in the microalgae). The copepods are able to alternate between the two mechanisms [24]. Grazing activities of zooplankton are also impacted by other factors, such as temperature and illumination [9]. Some species of bacteria, called phytoplankton-lytic bacteria, are able to inhibit the growth of microalgae and some of these species can cause mass destruction of the microalgae under cultivation. The attack depends on cell-to-cell contact or is mediated by extracellular compounds. Examples of such bacteria are *Alteromonas* sp., *Flavobacterium* sp., *Cytophaga* sp., *Myxobacter* sp., *Bacillus* sp., *Pseudomonas* sp., *Saprospira* sp. (SS98-5), and *Pseudoalteromonas* sp. 25 [9,25].

### Strategies for contamination control

**Figure 2:**
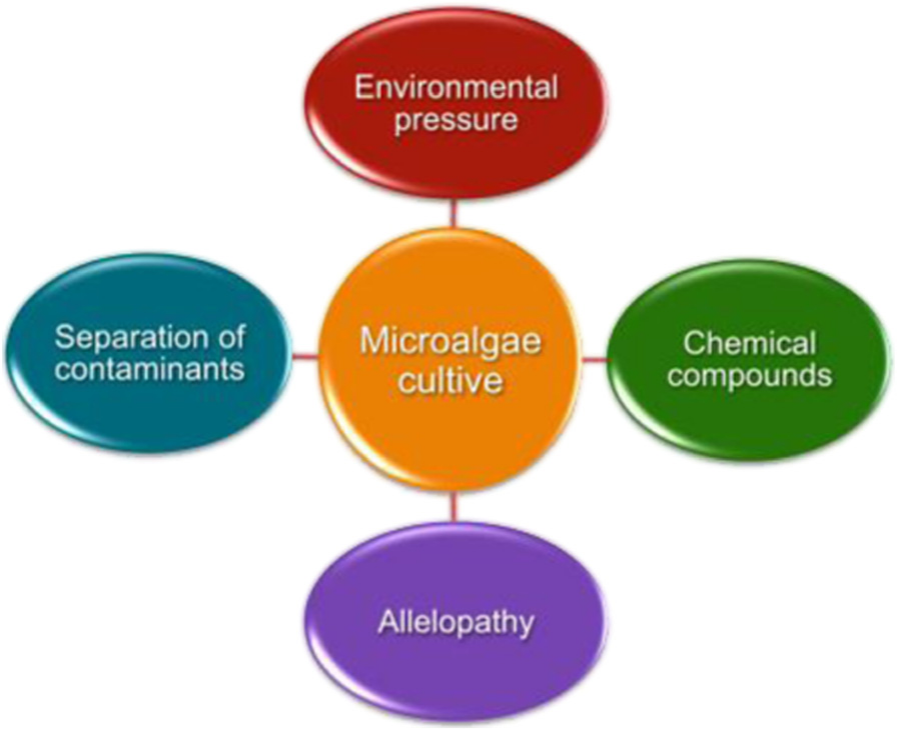
Strategies to control contaminants in microalgae cultivation.

Environmental pressure, chemical compounds, separation of contaminants, herbicides/pesticides and other chemical compounds are some of the strategies currently used to combat biological contamination and they are undergoing intense research and development worldwide (Figure 2). The establishment of a strong environmental pressure is a method widely used to control contaminants. Some microalgae are able to endure extreme conditions of cultivation and this may act as a competitive advantage in relation to contaminants in the liquid medium. An example is *Dunaliella salina*, which can grow in high concentrations of salinity, greater than 4% per mass [26]. The application of a specific stressor, such as a radical increase in the salinity of the microalgae medium, can damage the regulatory system of the predators without any loss of microalgae cells [14,20]. Reduction of the pH to 3.0 in a microalgae culture has been shown to kill flagellates [27]. Another method to combat biological contamination is to add chemical compounds that induce the biological contaminant to separate from the algal cells and it can then be concentrated in a special compartment of the photobioreactor from which it can be discarded.

Pesticides such as Trichlorphon, Decamethrin, Tralocythrin, and Buprofezin have also been used to annihilate zooplankton in microalgae suspensions [9]. Chemical compounds such as quinine sulfate and ammonia bicarbonate have been used to eliminate protozoa predators [28,29]. In some open-pond cultivation processes, an organic carbon substrate, such as acetate, is added continuously in small quantities in order to support higher biomass concentrations while it prevents excessive bacteria growth [30]. Microorganism contaminants can also be mechanically separated by size. Microalgae cells, which are normally smaller, are able to pass through a filter element that retains larger predators (e.g., rotifer contamination). Due to the relatively small size of microalgal cells, filtration can also be considered as an effective method to remove larger biological organisms, such as rotifers and copepods. Silk mesh screens have been used for this purpose, but rotifer eggs and the developing young individuals could not be removed completely [31].

### Allelopathy

Allelopathy is a biological phenomenon by which an organism produces one or more biomolecules that affect the growth, survival, and/or reproduction of other organisms. These biomolecules, which are mostly secondary metabolites, are known as allelochemicals and are produced by certain plants, algae, bacteria, coral, and fungi. These compounds can have beneficial (positive allelopathy) or detrimental (negative allelopathy) effects on the target organism. Allelopathic interactions are an important factor in determining species distribution and abundance within plant and plankton communities. Biomolecules with allelopathic functions have other ecological roles, such as chemical defense, nutrient chelation, and in plants, regulation of soil biota [32-34]. These molecules could be used as natural herbicides and in recent decades allelopathy has been the target of much research in order to apply this biological phenomenon to promote pest control in crops for sustainable agriculture [35-39]. Allelochemicals produced by plants are a potential source for alternative agrochemicals and have become a new strategy. Several examples have been described such as an inhibition of 100% in the growth of the weed *Gallium spurium* with plant extracts [40] and the inhibitory effect of essential oils in algae, molds, and yeasts [32,33]. Sorghum produces sorgoleone a lipidic benzoquinone, which is currently used as a herbicide in agriculture [41].

Allelopathy studies have been carried out with microalgae, mostly focusing on negative allelopathy. For example, the dinoflagellate *P. aciculiferum* negatively impacts *Synura petersenii* (Chrysophyceae), *Peridinium inconspicuum* (Dinophyceae), *Cyclotella* sp. (Bacillariophyceae), C*ryptomona*s sp., and *Rhodomonas lacustris* (Cryptophyceae) through lysis. The allelopathic activity of *P. aciculiferum* may result from the impact of a single chemical or a cocktail of allelochemical molecules [42]. Microalgae allelopathy may negatively impact the performance of predators, leading to their death or inactivation. The strategy has been widely shown in toxic algae during blooming [10], and is an interesting approach for predator control. The most promising studies are those in which microalgae feed on the predators through already proven complex mechanisms such as phagotrophym. It is possible that some toxins are involved in prey capture. For instance, it has been observed that some phytoplankton such as Dinoflagellates paralyze the prey before ingestion [34,43,44].

### Biotic and abiotic factors

Allelopathy can be stimulated or minimized by a number of biotic and abiotic factors. Among the more important abiotic factors that enhance and stimulate allelopathy are deficiency of nutrients such as nitrogen and phosphorous compounds in the culture medium, low light intensities, low temperatures, and a culture medium with a high pH (around 9.0). Abiotic factors acting as repressors of allelopathy include high light intensities, high temperatures, excessive nutrients in the culture medium (nitrogen and phosphorus), and culture medium with low pH values (pH around 6.0). The chemical structure of the toxic protein compounds and the mechanisms by which changes in abiotic factors stimulate or inhibit allelopathy have not yet been fully elucidated [45]. The most important biotic factors are the cellular concentrations of the microalgae producing toxic proteins, and the target cells. For example high concentrations of the microalgae *Heterocapsa circularisquama* and ciliates can bring about the death of the ciliates in an effect similar to “quorum sensing” [42,45,46]. *Peridinium gatunense* and the cyanobacteria *Microcystis* sp. have been shown to inhibit each other through allelopathy [47].

### Phagotrophy and osmotrophy

Allelopathy can be further accompanied by processes of phagotrophy (ingestion of prey) and osmotrophy (ingestion of organic molecules) by microalgae without deactivation of the photosynthetic system. Some types of predatory species of microalgae present in zooplankton may serve as a source of nutrients in the cultivation of microalgae strains carrying a genetic potential for allelopathy. Some studies have shown even higher rates of growth in microalgae when they use osmotrophy and phagotrophy mechanisms, suggesting an intense energy use which seems to be related to the heterotrophic metabolism that some microalgae carry out. Among the many dinoflagellates that are able to carry out phagotrophy are *Ceratium furca*[48]*, Dinophysis acuminate*[49]*, Gonyaulax polygramma*[50]*, *and *Alexandrium tamarense*[51].

### Major allelochemicals in microalgae

Allelochemicals involved in the interactions between aquatic microalgae have received more attention in recent years. Allelopathy is an important factor for explaining community structure, dynamics of the populations, chemical defense of microalgae against potential microbes and micro-grazers in aquatic habitats, over and above the competition with other organisms for physical space, light and nutrients. The chemical defense against potential predators and grazers includes the larvae of aquatic invertebrates. These bioactive metabolites from microalgae provide a competitive advantage via inter-specific and particularly negative (i.e., inhibitory) effects on growth, survival, and reproduction of the antagonist species. Besides this, they are a source of new antimicrobial agents, herbicides and biopesticides [52,53].

**Figure 3:**
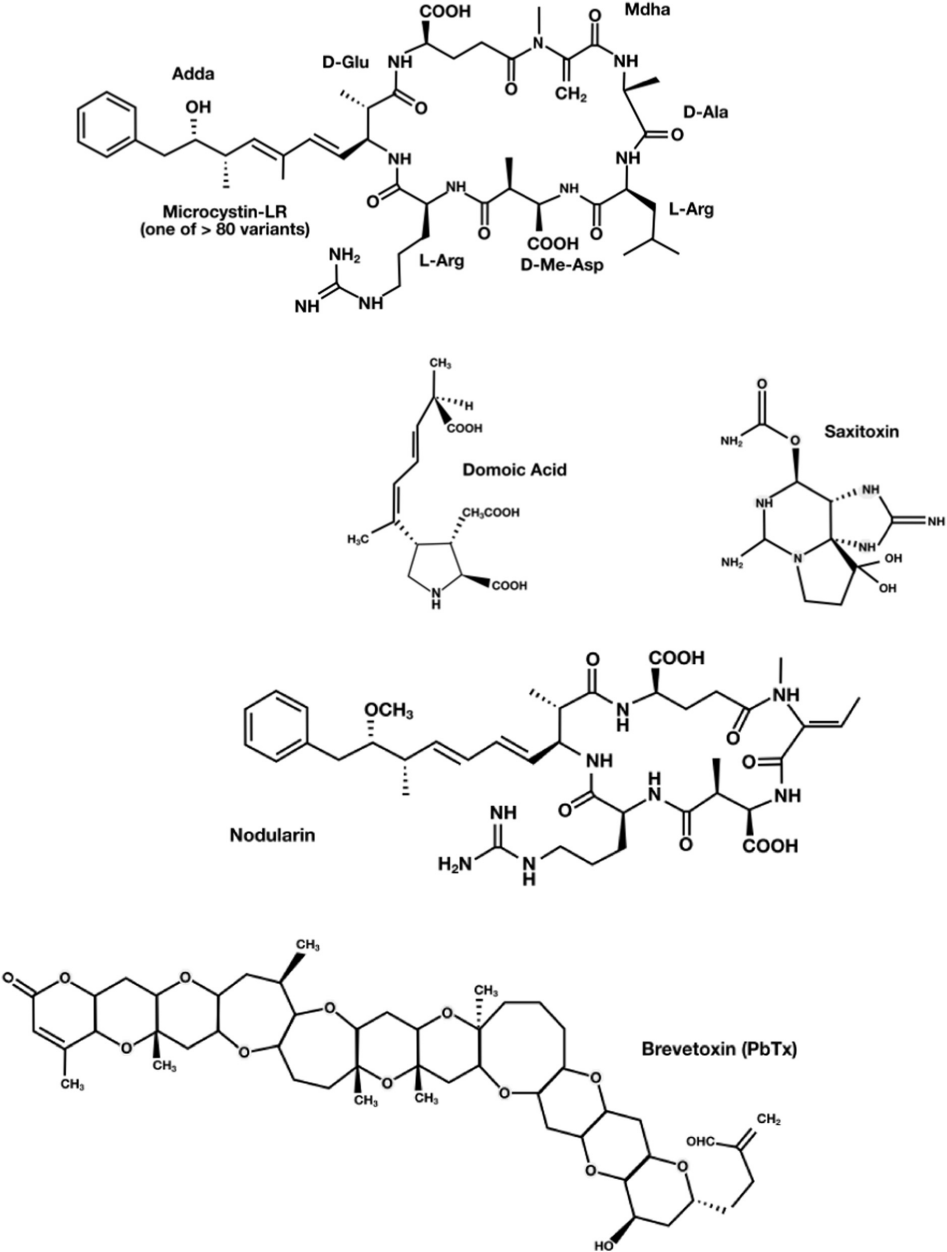
**Structures of toxins from marine and freshwater microalgae.** Based on Berry, 2011 [53]. Microcystin-LR is a cyclic heptapeptide produced by Cyanobacteria; Domoic acid is a structural analog of kainic acid and proline produced by Dinoflagellates; Saxitoxin is a neurotoxic alkaloid produced by dinoflagellates, Nodularin cyclic peptide is produced by the cyanobacterium *Nodularia* sp., and brevetoxin is represented by a cyclic polyether compound.

Microalgae produce a remarkable diversity of biologically active metabolites. Some allelochemicals from marine and freshwater microalgae are herbicidal compounds [52]. Secondary metabolites of the microalgae include every chemical class of natural product, ranging from fatty acids to alkaloids, as well as many peptides and amino acids [53,54]. The production of toxins by phytoplankton is also well known. Dinoflagellates can produce the toxin brevetoxin, a cyclic polyether compound, and Ciguatoxin, a carbon polycyclic compound that activates the Na^+^ channel. Studies have shown that *Karenia brevis*, which produces brevetoxin, can affect the fertility of a copepod predator [46,55,56]. However, it is not yet clear whether this effect is a direct cause of the exposure to the microalgae toxin or the result of the poor nutritional value of *K. brevis*[57]. Saxitoxin is a neurotoxic alkaloid that blocks the Na^+^ channel [58]. Domoic acid, which is a structural analog of kainic acid and proline, is produced by diatoms, and causes neuronal depolarization [59]. In some cases, heterotrophic bacteria can degrade toxins produced by phytoplankton [60] Another example is the cyanobacterium algae that produce the anatoxin-a (bicyclic secondary amine) and the microcystin-LR (cyclic heptapeptide) toxins that paralyze the motile green alga *Chlamydomonas*, leading to fast sedimentation of *Chlamydomonas*[45,61] (Figure 3). The metabolites belonging to the indole class of alkaloids have anti-algal activity, and consequently are associated with allelopathic interactions. The most cited allelochemicals from cyanobacteria are the hapalindoles and their related alkaloids [62,63].

Few studies have demonstrated the effects of microalgae allelopathic compounds against viruses. However, inhibitory activities of methanol extracts from the microalgae *Ankistrodesmus convolutus*, *Synechococcus elongatus*, and *Spirulina platensis* against Epstein-Barr virus (EBV) have been reported. The extracts reduced the cell-free EBV DNA, indicating that the lytic cycle or the release of the viruses could be inhibited [64]. Polysaccharides have been poorly investigated as allelopathic agents, both in aqueous and terrestrial ecosystems [36] but polysaccharides from *Navicula directa, Gyrodinium impudicum, Ellipsoidon* sp., Cryptomonads, *Cochlodinium polykrikoide* and a Pheophorbide like compound from *Dunaliella primolecta* have antiviral activities [65]. This section describes the major microalgae allelochemicals and Table 2 summarizes the major types with their major targets.

**Table 2:** Allelochemicals and target

**Compounds**	**Organism**	**Target**	**Reference**
**Phenolic compounds**			
4,4’-dihydroxybiphenyl	*Nostoc insulare Geitlerinema* sp.	Cyanobacteria Bacteria	[66,67]
		Fungi	
**Fatty acid**			
Timnodonic acid and stearidonic acid	Red algae : *Neodilsea yendoana*, *Palmaria palmata, Chondrus yendoi*, and *Ptilota filicina*	Eukaryotic microalgae red tide planktons (*Chattonella antiqua* )	[68,69]
α-linolenic, oleic, linoleic, and palmitic acids	*Botryococcus braunii*	Copepoda (*Nauplius* sp., *Eodiaptomus* sp., *Cyclopina* sp. Cladocera (*Bosmina* sp., *Diaphanosoma* sp.) Rotifera (*Keratella* sp).	[70]
**Alkaloids**			
Nostocarboline	*Nostoc* sp , *Nodularia* sp.	*Microcystis aeruginosa Kirchneriella contorta Synechococcus Gram- negative bacteria*	[71,72]
Dimers of Nostocarboline		Gram positive (methicillin-resistant *Staphylococcus aureus*	[73]
12-epi-hapalindole E isonitrile 12-epi-hapalindole F	*Fischerella* sp *Hapalosiphon* sp	*Escherichia coli Microcystis* (strains NPLS 1, NPJB 1 and CENA 62) *Synechococcus* PCC 7942.	[74,75]
Ambiguines Ambiguine H isonitrile and ambiguine I isonitrile)	*Fischerella sp.*	*Escherichia coli ESS K-12 Staphyloccocus albus a Bacillus subtilis a Saccharomyces cereisiae Candida albicans*	[76]
Calothrixin	*Calaothrix* sp	*Bacillus subtilis Plasmodium falciparum*	[74]
Fischerellins A	*Fischerella* sp	*Synechococcus* PCC 7942.	[75]
Welwitindolinones	*Hapalosiphon welwitschii Westiella intricata*	Antifungal and insecticidal activity	[77]
**Peptides**			
microcystin-LR	*Cyanobacteria*	*Chlamydomonas reinhardtii,*	[61]
**Polyunsaturated aldehydes (PUAs)**			
Decatrienal	Diatom (*Thalassiosira* sp.)	Copepods Phytoplankton Bacteria	[78]
Octatrienal and Heptadienal	Diatomn (Skeletonema marinoi *Thalassiosira* sp)	Copepods Phytoplankton	
**Terpenoids**			
Comnostins	*Nostoc commune*	Antibacterial activity against *Staphylococcus epidermis*	[79]

#### Aldehydes

Oxylipins are derived from the oxidation of fatty acid. Polyunsaturated aldehydes (PUAs) are the most toxic oxylipins and were first discovered in marine and freshwater diatoms. Other oxylipins are hydroxy acids and epoxy alcohols but the PUA decadienal is the most studied PUA. The molecule has an effect on the grazer defense of diatoms against copepods [78]. The PUAs 2E,4E-decadienal, 2E,4E-octadienal and 2E,4E-heptadienal, have a toxic allelopathic effect on chlorophyte *Tetraselmis suecica*, the diatom *Skeletonema marinoi* and on the dinoflagellate *Amphidinium carterae*[80]. In diatoms, aldehyde (2E,4E/Z)-decadienal induced a process of cellular signaling. Decadienal can trigger intracellular calcium transients and generate nitric oxide (NO) by a calcium-dependent NO synthase-like activity, resulting in cell death [81]. Besides this, antibacterial activity of these compounds has been described by [82].

#### Glycolipids and fatty acids

Allelopathic compounds have been described for the green algae *Botryococcus braunii*, favoring its dominance in its natural environment. The compounds are a mixture of free fatty acids including α-linolenic, oleic, linoleic, and palmitic acids. The fatty acids become toxic to phytoplankton only when they are liberated by *B. braunii* to extracellular medium. In habitats with a pH between 8.0 and 9.0 fatty acids exist in a free form, namely RCOO^-^, a form known to be more toxic to aquatic organisms than uncharged forms inhibiting electron transport in chloroplasts [70,83]. Phytoplankton and zooplankton including Diatoms, Dinoflagellates, Chrysophyceae, red and brown algae produce fatty acids. Other effects such as membrane disruption and formation of oxidation product, antimicrobial activity, secondary messengers for biochemical pathways, and phospholipase action have been reported [84,85].

Sulfated galactosyl (i.e. sulfoquinovosyl) a glycolipid from the thylakoid membranes of prokaryotic and eukaryotic photoautotrophs has inhibitory activity to eukaryotic DNA polymerase and is an anti-cancer agent [86].

#### Phenolic compounds

Phenolic compounds have substantial allelopathic applications in agriculture and forestry as herbicides, insecticides, and fungicides [87], Some aquatic plants are algaecidal such as as *Schoenoplectus lacustris*[88] and *Myriophyllum spicatum* producing respectively, benzoic and protocatechuic acid and Gallic, ellagic and pyrogallic acids. The effects of these phenolic compounds have been studied to control the algal bloom. It is interesting to note that the allelopathic compounds show spectra of action specific to determined groups of organisms allowing the modulation of their effects and action [89].

#### Alkaloids

A number of metabolites belonging to the indole class of alkaloids have been found to possess anti-algae activity, associated with allelopathy. Indeed, the most frequently cited allelochemicals from cyanobacteria are the hapalindoles and related alkaloids: 12-epi-hapalindole and isonitrile, including ambiguines, welwitindolinones and fischerindoles that have been isolated from both marine and freshwater representatives of the family Stigonemataceae, and particularly the genera *Hapalosiphon* sp. and *Fischerella* sp. [53].

#### Oligopeptides and cyclic peptides

Microcystins produced by the cyanobacteria are cyclic hepta-hepatopeptides belonging to the group of cyanotoxins. Extracellular products made by the cyanobacterium *Anabaena flos-aquae* contained both anatoxin and microcystin, and significantly reduced the growth of *Chlamydomonas reinhardtii*, a green alga. Besides this, high concentrations of *C. reinhardtii* extracellular products completely inhibited microcystin accumulation. These results demonstrate that cyanobacterial toxin production may be regulated by a complex mechanism involving growth phase dependence and environmental conditions [61] (Another compound is Portoamide which is a cyclic peptide produced by *Chlorella vulgaris*. Thus metabolites differentially inhibited some cyanobacteria, including *Cylindrospermopsis raciborskii.* Other cyanobacteria such as *Microcystis* sp, *Aphanizomenon* sp and *Anabaena* sp. and microalgae (e.g. the diatom*, Cyclotella menenghiniana*) tested were not inhibited [90].

#### Lactones

Cyanobacterin is a chlorinated γ–lactone, from *Scytonema,* a genus of photosynthetic cyanobacteria, which specifically inhibited a range of microalgae, including cyanobacteria and green algae, at micromolar concentrations [91]. The action mechanisms is the inhibition of photosystem II [92].

### Inhibitory effects of allelochemicals

#### Enzyme inhibition

Enzymes are essential for all organisms catalyzing multiple functions. Microalgae including cyanobacteria produce glycosidase and peptidase inhibitors [93]. Researchers in Taiwan described that 20% of the cyanobacteria isolates from biofilms possess a glucosidase inhibitory activity, an allelopathic activity and grazer toxicity [94]. The inhibitors are hydrolyzable polyphenols which complex with proteins, inhibiting the enzymes [95]. An extracellular, low molecular weight α -amylase inhibitor was isolated from *Anabaena.* Tannins are stored in vacuoles of filamentous chlorophyte, and can inhibit activities of peroxidase (POD), catalase and cellulose [93,96]. Phenolic allelochemicals such as chlorogenic acid, caffeic acid and catechol can inhibit activities of phosphorylase; cinnamic acid and its derivatives can inhibit the hydrolysis activities of ATPase [97].

#### RNA synthesis, DNA replication and protein synthesis

The alkaloids 12-epi-hapalindole E isonitrile and Calothrixin A are algicidal metabolites isolated respectively from the cyanobacterial *Fischerella* sp and *Calaothrix* sp. (Table 1). Both of them have been demonstrated to inhibit the RNA synthesis, and consequently protein synthesis, in intact cells of *Bacillus subtilis.* These compounds inhibited *Escherichia coli* RNA polymerases directly. The indolophenanthridine calothrixin A from cyanobacterium *Calothrix* species inhibited DNA replication [74].

#### Photosynthesis

The cyanobacteria *Fischerella muscicola* produces fischerellin A (Table 2), a toxic allelochemical compound and potent photosystem-II inhibitor acting against other cyanobacteria and photoautotrophic organisms [98]. The crude lipophilic extracts containing indole alkaloids from *Fischerella* sp. strain 52 inhibit photosynthesis of the green alga *Chlamydomonas* sp. in a concentration- and time-dependent manner, causing loss of ultra-structural cell organization [62]. The aquatic monocotyledonous angiosperm, *Lemna minor* has been utilized as a model system for algae derived herbicides. *Lemna minor* was used to identify herbicidal compounds from ethanol extracts of the cyanobacterial species, *Lyngbya aesturii*. The herbicidal activity was due to the fatty acid, 2,5-dimethyldodecanoic acid which inhibited growth at a concentration of 200 ng/mL [99]. In *lemma minor* Microcystin inhibited growth, photosynthesis and oxidative stress [100].

#### Toxin-assisted micropredation

This phenomenon was observed in blooms of *Prymnesium parvum,* a flagellated alga. These microalgae have caused massive killings of fish around the world due to the release of allelochemicals. The study demonstrated the effects of *Prymnesium* on grazing zooplankton. Toxins are released during cell-to cell-contact in a mechanism called “toxin-assisted micropredation” [101].

### Allelochemicals for the industrial cultivation of microalgae

Microalgae have many applications in biotechnology, biofuels, pharmaceuticals, the food industry and aquaculture [9]. Microalgae, or their derived-products, are also used in cosmetics and biofertilizers, in protein-rich animal feeds and chemical feedstocks [102]. The limiting factor for biodiesel production in open ponds with microalgae is contamination. New cultivation technologies have to be focused for industrial purposes using microalgae. The need for a biological method that is environmentally friendly for contamination management is increasing due to the problems associated with the constant and intensive use of chemical herbicides [39]. Besides this, natural herbicides exhibit a wider range of target sites than synthetic inhibitors. Studies with allelochemicals and their applications show the potential of natural products to be used to minimize contamination in microalgae cultures. It is a promising route for future applications. Further investigations need to be made in order to develop this important topic relative to the use of allelochemical compounds. At the present moment the cyanobacteria allelochemicals and algal toxins have been studied due to economic questions caused by the toxic algae bloom (HAB). In this context, allelopathy mediated by allelochemicals is an area with a wide range of biotechnological applications [35,53]. Another interesting use of bioactive compounds from microalgae is in biofouling. Biofouling is one of the more serious problems currently in maritime domains and affects the biofuels sector directly. Several substances with antifouling activity have been isolated from microalgae (mainly from cyanobacteria) such as fatty acids, lipopeptides, amides, alkaloids, terpenoids, lactones, pyrroles and steroids [103]. An example is the Cyanobacterin isolated from *Scytonema hofmanni,* which has been found to deter populations of the fouling benthic diatom *Nitzschia pusilla*[103]. The Ent-labdane diterpenes that are low polar compounds, could possibly play an ecological role such as antifouling compounds [36].

### Allelopathy: a potential novel strategy for contamination control

Millions of liters of cultivation are lost due to biological contamination. In most cases, predators belonging to zooplankton grow rapidly, causing irreparable damage to the cultivation [10]. Problems concerning the incidence of fungi were reported in closed photobioreactors for the culture *Haematococcus fluvialis*. The solution was the addition of specific carbohydrates to the culture medium to compete with the preferred binding site of the fungus, a glycolipid found on the cell wall of the microalgae [104]. In the case of open ponds, significant losses of algae cultivation have been observed in a period of days or even hours. The researchers engaged in the combat and control of this type of situation must have specific training in algae culturing and be exceptionally well prepared. In a presentation at the "Algae Biomass Summit" in 2011, Hu highlighted that biological contamination may have a devastating effect on the population dynamics of microalgae, destroying tons of culture in mere days.” In only six days the culture might be completely deteriorated by rotifers, ciliates, and vorticella” (Hu, 2011, personal communication). Using microalgae strains with a high capacity to combat predators and parasites is an extremely interesting possibility for scaling up processes carried out in open ponds, because the potential for failure or “crashing” of the crop production would be reduced. This possibility alone would fully justify the investments needed to better elucidate the mechanisms of allelopathy. An understanding of the biochemical communication processes involved in the microbial ecology of open ponds might allow us to find mechanisms to control contamination in algae cultivation [9,20].

**Figure 4:**
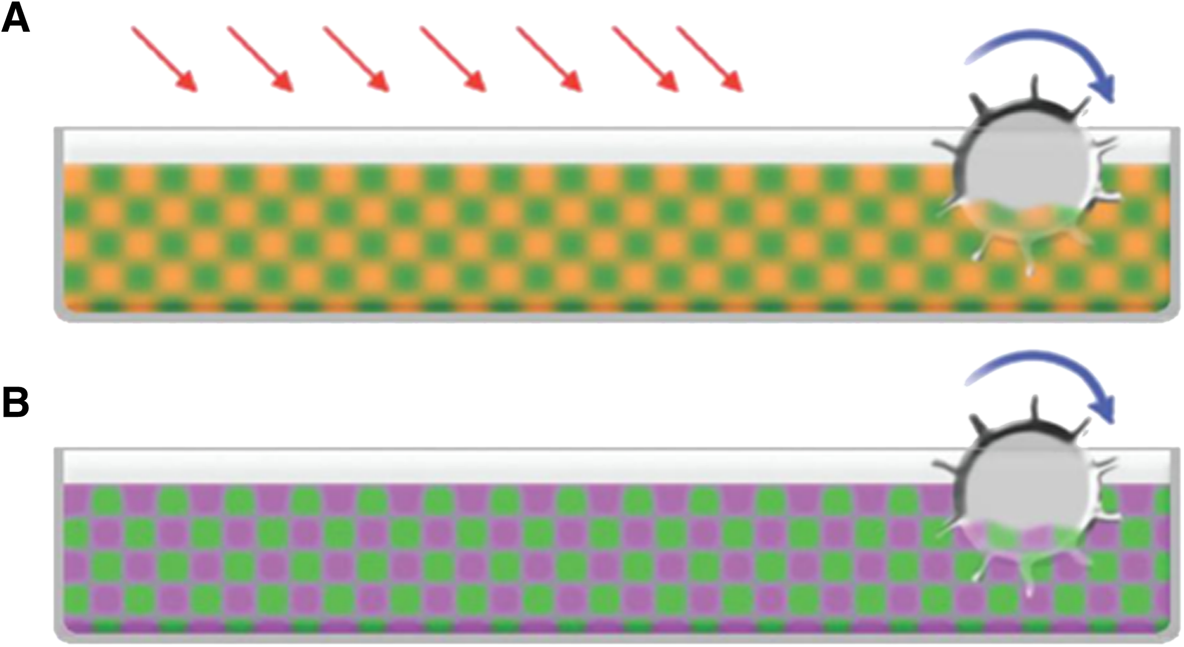
**Control of biological contamination. A**. Chemicals (red arrows) are released into the culture medium in order to combat biological contamination (orange) in an open system containing microalgae (green).The circle represents the blades turning at low speed for system circulation. **B**. Controlled management of allelopathy-derived compounds (magenta) from microalgae (green) eliminating biological contamination in open-pond cultivation. The circle represents the blades rotating at low speed for system circulation.

One of the classical approaches currently used and recommended by several authors to combat and prevent losses in algae cultivation includes adding specific chemical compounds (e.g., sodium hypochlorite) to the medium in order to combat predators (such as Protozoa). The use of biocide substances increases the cost in large-scale cultivation [15] (Figure 4). However, this approach entails additional financial costs and is labor-intensive. A controlled approach that triggers allelopathy can be designed. Such a system would not give much opportunity for predation of the microalgae since the microalgae of interest would themselves eliminate the competitors and predators by feeding on them through phagotrophy in conditions of lack of nutrients in the culture medium (less nitrogen and phosphate compounds). This strategy would result in an improved productivity and possibly financial savings due to the need to add fewer nutrients to the medium. In this case, deactivation of potential toxins in the culture medium through photo-oxidation or other processes before launching the culture medium into a disposal reservoir would be essential for biosafety and environmental protection. This innovative allelopathic approach could also draw on transgenic techniques to have genes inserted in microalgae strains of interest, making cultivation possible in the face of self-defense against competitors and predators. The allelopathic strategy has not yet been fully explored. Additionally, it requires rigorous control based on the laws and regulations of each country in order not to become a threat to the environment.

In Brazil, a potential strategy to help control biological contamination in algae cultivation would be the release of allelopathic compounds extracted from microalgae biomass (e.g., filtered extract of cyanobacteria) that occur naturally as harmful blooms impacting water quality in some regions of the country (Panosso, 2012, personal communication). However, for this strategy to be implemented, a very strict control process would be necessary due to the toxicity of these compounds.

**Figure 5:**
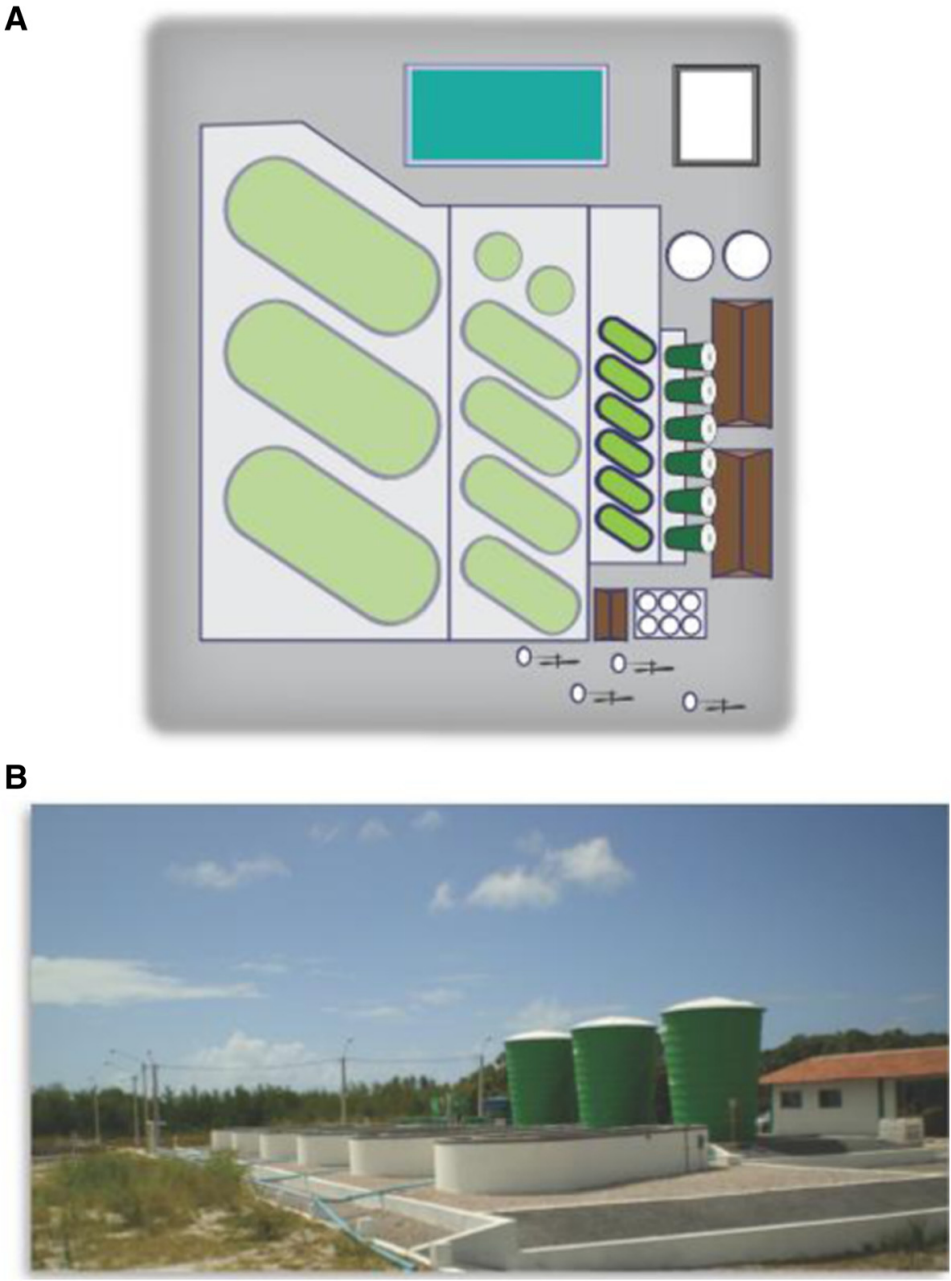
**Microalgae pilot plant. A**. Schematic diagram of the microalgae pilot plant for the production of biodiesel. At present the plant has the capacity to operate open ponds with a unit volume of 6,000 liters **B**. Photograph of the microalgae pilot plant for the production of biodiesel located in Extremoz at the Aquaculture Technology Center of UFRN (Universidade Federal do Rio Grande do Norte). Author’s personal file, 2013.

CENPES-PETROBRAS (Petrobras Research Center, Brazil), in partnership with the Federal University of Rio Grande do Norte (UFRN) in Natal, Brazil, has been growing microalgae successfully on a pilot scale for biodiesel production since 2009 (Figure 5A,B). Currently, the capacity to operate open ponds that have a total area of 100 m^2^, producing 6,000 liters of algae culture has been achieved. The complete plant was launched in 2012 with the goal to produce sufficient microalgae biomass for biodiesel production, using native strains. Research into contamination control using different approaches has been the subject of R&D at CENPES-PETROBRAS and includes studies of the allelopathic processes in microalgae cultivation. This project is essential for the scientific and technological development of the Northeast region. Quadrant winds and high luminosity throughout the year have allowed a successful production of microalgae biomass, ensuring high productivity in open ponds all year round. The biomass collected at this plant is stored at low temperatures before undergoing the extraction process and conversion into biodiesel.

## Conclusions

The control of biological contamination is currently identified as one of the greatest obstacles to achieving positive results in the cultivation of microalgae in both open and closed systems. To scale up the cultivation of microalgae more data are needed on the mechanisms that stimulate or inhibit the production processes of allelopathic compounds by microalgae in cultivation systems, at lab, pilot, and commercial production scales. The development of this line of research could lead to innovative methods of cultivation in which low quantities of nutrients are needed and a strict control of predators is achieved in a more economical manner.

Apart from the data produced through research projects conducted in the lab, the scientific literature on the specific topic of allelopathy for microalgae cultivation is scarce. Thus, greater R&D investments in this field of biotechnology are needed.

Screening for, and selection, isolation, and maintenance of new microalgae species that are adequate for use with allelopathy strategies is of great importance for future studies to provide a better understanding of the causes and mechanisms involving predator and prey in microalgae cultivation systems, whether for the production of biofuels or other chemical compounds of interest. This is an Emerging Field but with a great potential for microalgae cultivation for biodiesel and other industrial applications.

## Abbreviations

EBV: Epstein-Barr virus; PUAs: Polyunsaturated aldehydes; NO: Nitric oxide; POD: Peroxidase; HAB: Toxic algae bloom; PbTx: Brevidtoxin.

## Competing interests

The authors declare that they have no competing interests.

## Authors’ contribution

This review was written by LBBM and ABV. Both authors read and approved the final manuscript.

## References

[B1] Brennan L Owende P Biofuels from microalgae—A review of technologies for production, processing, and extractions of biofuels and co-products Renew Sustain Energy Rev 2010 6 2 557 577

[B2] Rawat I Ranjith- Kumar R Mutanda T Bux F Dual role of microalgae: Phycoremediation of domestic wastewater and biomass production for sustainable biofuels production Appl Energy 2011 6 3411 3424

[B3] Mutanda T Ramesh D Karthikeyan S Kumari S Anandraj A Bux F Bioprospecting for hyper-lipid producing microalgal strains for sustainable biofuel production Bioresour Technol 2011 6 1 57 70 20624676 10.1016/j.biortech.2010.06.077

[B4] Liu J Huang J Chen F Stoytcheva M Microalgae as feedstocks for biodiesel production Biodiesel - Feedstocks and Processing Technologies 2011 Croatia: InTech 58 78

[B5] Mata TM Martins AA Caetano NS Microalgae for biodiesel production and other applications: a review Renew Sust Energ Rev 2010 6 217 232

[B6] Lam MK Lee KY Microalgae biofuels: A critical review of issues, problems and the way forward Biotechnol Adv 2012 6 3 673 690 22166620 10.1016/j.biotechadv.2011.11.008

[B7] Dassey AJ Theegala CS Harvesting economics and strategies using centrifugation for cost effective separation of microalgae cells for biodiesel applications Bioresour Technol 2012 6 241 245 23196245 10.1016/j.biortech.2012.10.061

[B8] Halim R Danquah MK Webley PA Extraction of oil from microalgae for biodiesel production: A review Biotechnol Ad 2012 6 3 709 732 10.1016/j.biotechadv.2012.01.001 22266377

[B9] Wang H Zhang W Chen L Wang J Liu T The contamination and control of biological pollutants in mass cultivation of microalgae Bioresour Technol 2013 6 745 750 10.1016/j.biortech.2012.10.158 23186675

[B10] Tillmann U Interactions between Planktonic Microalgae and Protozoan Grazers J Eukaryot Microbiol 2004 6 2 156 168 15134250 10.1111/j.1550-7408.2004.tb00540.x

[B11] Granéli E Turner JT Caldwell MM, Heldmaier G, Jackson RB, Lange OL, Levia DF, Mooney HA, Schulze E-D, Sommer U Ecology of harmful algae Ecological Studies 2006 189 Germany: Springer Berlin Heidelberg 1 413

[B12] Graham JE Wilcox LW Graham LE Algae 2008 San Francisco, CA: Benjamin Cummings (Pearson) 1 790

[B13] Lardon L Hélias A Sialve B Steyer JP Bernard O Life-cycle assessment of biodiesel production from microalgae Environ Sci Technol 2009 6 6475 6481 19764204 10.1021/es900705j

[B14] Hu Q Zarmi Y Richmond A Effects of light intensity, light path and culture density on output rate of *Spirulina platensis* (Cyanobacteria) Eur J Phycol 1998 6 165 171

[B15] Klein-Marcuschamer D Chisti Y Benemann JR Lewis D A matter of detail: assessing the true potential of microalgal biofuels Biotechnol Bioeng 2013 6 9 2317 2322 23733523 10.1002/bit.24967

[B16] MaBorowitzka L Pauw N, Persoone G **Micro-algal biotechnology** Micro-Algal Biotechnology 1988 Cambridge: Cambridge University Press 197 221

[B17] Patil V Tran KQ Giselrød HR Towards sustainable production of biofuels from microalgae Int J Mo Sci 2008 6 1188 1195 10.3390/ijms9071188 PMC2635721 19325798

[B18] Van Etten J Dunigan DD Chloroviruses not your everyday plant virus Trends Plant Sci 2012 6 1 1 8 22100667 10.1016/j.tplants.2011.10.005 PMC3259250

[B19] Day JG Thomas NJ Achilles-Day U Leakey RJ Early detection of protozoan grazers in algal biofuel cultures Bioresour Technol 2012 6 715 719 22464416 10.1016/j.biortech.2012.03.015

[B20] Borowitzka MA Commercial production of microalgae: ponds, tanks, tubes and fermenters J Biotechnol 1999 6 313 321

[B21] Rosetta CH McManus GB Feeding by ciliates on two harmful algal bloom species, *Prymnesium parvum* and *Prorocentrum minimum* Harmful Algae 2003 6 2 109 126

[B22] Lurling M Beekman W Influence of food-type on the population growth rate of the rotifer *Brachionus calyciflorus* in short chronic assays Acta Zool Sinica 2006 6 1 70 78

[B23] Frederiksen M Edwards M Richardson AJ Halliday NC Wanless S From plankton to top predators: bottom-up control of a marine food web across four trophic levels J Animal Ecol 2006 6 6 1259 1268 10.1111/j.1365-2656.2006.01148.x 17032358

[B24] Vanderploeg HA Paffenhofer G Models of algal capture by the freshwater copepod *Diaptomus sicilis* and their relation to food-size selection Limnol Oceanogr 1985 6 871 885

[B25] Shi SY Liu YD Shen YW Li GB Li DH Lysis of *Aphanizomenon folsaquae* (Cyanobacteria) by a bacteria *Bacillus cereus* Biol Control 2006 6 345 351

[B26] Shariati M Hadi MR Carpi A Microalgal biotechnology and bioenergy in *Dunaliella* Progress in Molecular and Environmental Bioengineering – From Analysis and Modeling to Technology Applications 2011 Croatia: InTech 483 505

[B27] Liu ZG Lu GL The sterilizing studies of flagellate and ciliate in marine unicellular algae liquid Zhanjiang Aquacult. Coll 1990 6 36 41

[B28] Moreno-Garrido I Canäavate JP Assessing chemical compounds forcontrolling predator ciliates in outdoor mass cultures of the green algae *Dunaliella salina* Aquacult Eng 2001 6 107 114

[B29] Méndez C Uribe E Control of *Branchionus* sp. and *Amoeba* sp. in cultures of Arthrospira sp Latin Am J Aquat Res 2012 6 3 553 561

[B30] Lee YK Microalgal mass culture systems and methods: Their limitation and potential J Appl Phycol 2001 6 307 315

[B31] Borowitzka MA Andersen RA Culturing microalgae in outdoor ponds Algal Culturing Techniques 2005 New York: Academic Press 205 217

[B32] Yang X Deng S De Philippis R Chen L Hu C Zhang W Chemical composition of volatile oil from *Artemisia ordosica* and its allelopathic effects on desert soil microalgae, *Palmellococcus miniatus* Plant Physiol Biochem 2012 6 153 158 22153252 10.1016/j.plaphy.2011.10.019

[B33] Inderjit Wardle DA Karban R Callaway RM The ecosystem and evolutionary contexts of allelopathy Trends Ecol Evol 2011 6 12 655 662 21920626 10.1016/j.tree.2011.08.003

[B34] Stoecker D Tillmann U Granéli E Granéli E, Turner JT Phagotrophy in Harmful Algae Ecological Studies, Ecology of Harmful Algae 2006 189 Heidelberg: Springer Berlin 177 187

[B35] Macias FA Marin D Oliveros-Bastidas A Varela RM Simonet AM Carrera C Molinillo JM Allelopathy as a new strategy for sustainable ecosystems development Biol Sci Space 2003 6 1 18 23 12897457 10.2187/bss.17.18

[B36] Macías FA Molinillo JM Varela RM Galindo JC Allelopathy-a natural alternative for weed control Pest Manag Sci 2007 6 4 327 348 17348068 10.1002/ps.1342

[B37] Farooq M Bran K Cheema ZA Wahid A Siddique KH The role ofallelopathy in agricultural pest management Pest Manag Sci 2011 6 5 493 506 21254327 10.1002/ps.2091

[B38] Albuquerque MB Santos RC Ima LM Filho PAM Nogueira RJMC Camara CAG Ramos AR Allelopathy, an alternative tool to improve cropping systems. A review Agron Sustain Dev 2011 6 379 395

[B39] Sodaeizadeh H Hosseini Z Allelopathy and environmentally friendly method for weed control International Conference on Applied Life Sciences (ICALS) 10–12 September 2012 2012 Turkey: In tech

[B40] Uddin MR Park KW Pyon JY Park S Combined herbicidal effect of two natural products (sorgoleone and hairy root extract of *tartary buckwheat*) on crops and weeds Aust J Crop Sci 2013 6 2 227 233

[B41] Santos ILVL Silva CRC Santos SL Maia MMD Sorgoleone: benzoquinona lipídica de sorgo com Efeitos alelopáticos na agricultura como herbicida Arq Inst Biol 2012 6 1 135 144

[B42] Rengefors K Legrand C Broad allelopathic activity in *Peridinium aciculiferum* Eur J Phycol 2007 6 4 341 349

[B43] Hansen PJ Calado AJ Phagotrophic mechanisms and prey selection in free-living dinoflagellates J Eukaryot Microbiol 1999 6 382 389

[B44] Roberts EC Legrand C Steinke M Wootton EC Mechanisms underlying chemical interactions between predatory planktonic protists and their prey J Plankton Res 2011 6 6 833 842

[B45] Granéli E Edvardsen B Roelke DL Hagström JA The ecophysiology and bloom dynamics of *Primnesium* spp Harmful Algae 2012 6 260 270

[B46] Turner JT Tester PA Hansen PJ Anderson D, Cembella AD, Hallegraeff M Interactions between toxic marine phytoplankton and metazoan and protistan grazers Physiological Ecology of Harmful Algal Blooms 1998 Germany: Springer Heidelberg 3 7

[B47] Vardi A Schatz D Beeri K Motro U Sukenik A Levine A Kaplan A Dinoflagellate-cyanobacterium communication may determine the composition of phytoplankton assemblage in a mesotrophic lake Curr Biol 2002 6 1767 1772 12401172 10.1016/s0960-9822(02)01217-4

[B48] Smalley GW Coats DW Stoecker DK Feeding in the mixotrophic dinoflagellate *Ceratium furca* is influenced by intracellular nutrient concentrations Mar Ecol Prog Ser 2003 6 137 151

[B49] Jacobson DM Anderson DM Widespread phagocytosis of ciliates and other protists by marine mixotrophic and heterotrophic thecate dinoflagellates J Phycol 1996 6 279 285

[B50] Jeong HJ Yoo YD Seong KA Kim JH Park JY Kim S Lee SH Ha JH Yih WH Feeding by the mixotrophic red-tide dinoflagellate *Gonyaulax polygramma*: mechanisms, prey species, effects of prey concentration, and grazing impact Aquat Microb Ecol 2005 6 249 257

[B51] Jeong HJ Yoo YD Park JY Song JY Kim ST Lee SH Kim KY Yih WH Feeding by the phototrophic red-tide dinoflagellates: 5 species newly revealed and 6 species previously known to be mixotrophic Aquat Microb Ecol 2005 6 133 150

[B52] Macías FA Galindo JLG Garcia-Diaz MD Galindo JCG Allelopathic agents from aquatic ecosystems: potential biopesticides models Phytochem Rev 2007 6 155 178

[B53] Berry J Kortekamp A Marine and freshwater microalgae as a potential source of novel herbicides Herbicides and Environment 2011 Croatia: In tech 705 734

[B54] Jones AC Gu L Sorrels CM Sherman DH Gerwick WH New tricks from ancient algae: natural products biosynthesis in marine cyanobacteria Curr Opin Chem Biol 2009 6 216 223 19307147 10.1016/j.cbpa.2009.02.019 PMC2706127

[B55] Collumb CJ Buskey EJ Steidinger KA, Landsberg JH, Tomas CR, Vargo GA Effects of the toxic red tide dinoflagellate (*Karenia brevis)* on survival, fecal pellet production and fecundity of the copepod *Acartia tonsa* 2004 Petersburg: Florida Fish and Wildlife Commission Harmful Algae 2002

[B56] Paul VJ Arthur KE Ritson-Williams R Ross C Sharp K Marine biological laboratory chemical defenses: from compounds to communities Biol Bul 2007 6 226 251 10.2307/25066642 18083964

[B57] Breie CF Buskey EJ Effects of the red tide dinoflagellate, Karenia brevis, on grazing and fecundity in the copepod *Acartiatonsa* J Plankton Res 2007 6 115 126

[B58] Wiese M D'Agostino PM Mihali TK Moffitt MC Neilan BA Neurotoxic alkaloids: saxitoxin and its analogs Mar Drugs 2010 6 7 2185 2211 20714432 10.3390/md8072185 PMC2920551

[B59] Turner JT Tester PA Toxic marine phytoplankton, zooplankton grazers, and pelagic food webs Limnol Oceanogr 1997 6 1203 1214

[B60] Hulot FD Huisman J Allelopathic interactions between phytoplankton species: the roles of heterotrophic bacteria and mixing intensity Limnol Oceanogr 2004 6 4 1424 1434

[B61] Kearns KD Hunter MD Toxin-producing Anabaena flos-aquae induces settling of *Chlamydomonas reinhardtii*, a competing motile alga Microb Ecol 2001 6 80 86 12035083 10.1007/s002480000086

[B62] Gantar M Berry JP Thomas S Wang M Perez R Rein KS King G Allelopathic activity among cyanobacteria and microalgae isolated from Florida freshwater habitats FEMS Microbiol Ecol 2008 6 55 64 18266743 10.1111/j.1574-6941.2008.00439.x PMC2576510

[B63] Leão PN Vasconcelos MT Vasconcelos VM Allelopathy in freshwater cyanobacteria Crit Rev Microbiol 2009 6 271 282 19863381 10.3109/10408410902823705

[B64] Kok YY Chu WL Phang SM Mohamed SM Naidu R Lai PJ Ling SN Mak JW Lim PKC Balraj P Khoo ASB Inhibitory activities of microalgal extracts against Epstein-Barr virus DNA release from lymphoblastoid cells Zhejiang Univ Sci B 2011 6 5 335 345 10.1631/jzus.B1000336 PMC3087089 21528487

[B65] Amaro HMA Guedes C Malcata FX Méndez-Vilas A Antimicrobial activities of microalgae: an invited review Science against Microbial Pathogens: Communicating Current Research and Technological Advances **3** 2011 Spain: FORMATEX Microbiology Series 1272 1284

[B66] Volk RB Furkert F Antialgal, antibacterial and antifungal activity of two metabolites produced and excreted by cyanobacteria during growth Microbiol Res 2006 6 180 186 16427523 10.1016/j.micres.2005.08.005

[B67] Caicedo NH Kumirska J Neumann J Stolte S Thöming J Detection of bioactive exometabolites produced by the filamentous marine Cyanobacterium *Geitlerinema* sp J Mar Biotechnol 2012 6 436 445 10.1007/s10126-011-9424-1 PMC3374093 22160344

[B68] Suzuki Y Takabayashi T Kawaguchi T Matsunaga K Isolation of an allelopathic substance from the crustose coralline algae, *Lithophyllum* spp., and its effect on the brown alga, *Laminaria religiosa* Miyabe (Phaeophyta J Exp Mar Biol Ecol 1998 6 69 77

[B69] Kakisawa H Asari F Kusumi T Toma T Sakurai T Ohusa T Hara Y Chihara M An allelopathic fatty acid from the brown alga *Cladoshiphon okamuranus* Phytochemistry 1988 6 731 735

[B70] Chiang I-Z Huang W-Y Wu J-T Allelochemicals of *Botryococcus braunii* (Chlorophyceae) J Phycol 2004 6 474 480

[B71] Volk RB Screening of microalgal culture media for the presence of algicidal compounds and isolation and identification of two bioactive metabolites, excreted by the cyanobacteria *Nostoc insulare* and *Nodularia harveyana*, respectively J Appl Phycol 2005 6 339 347

[B72] Blom JF Blom JF Brütsch T Barbaras D Bethuel Y Locher HH Hubschwerlen C Gademann K Potent algicides based on the cyanobacterial alkaloid nostocarboline Org Lett 2006 6 4 737 740 16468755 10.1021/ol052968b

[B73] Locher HH Ritz D Pfaff P Gaertner M Knezevic A Sabato D Schroeder S Barbaras D Gademann K Dimers of nostocarboline with potent antibacterial activity Chemotherapy 2010 6 4 318 324 20714150 10.1159/000320033

[B74] Doan TN Rickards RW Rothschild JM Smith GD Inhibition of bacterial RNA polymerase by the cyanobacterialmetabolites 12-epi-hapalindole E isonitrile and calothrixin A FEMS Microbiol Lett 2001 6 135 139 11267769 10.1111/j.1574-6968.2001.tb10554.x

[B75] Etchegaray A Rabello E Dieckmann R Moon DH Fiore MF H von Dohre H Tsai SM Neilan BA Algicide production by the filamentous cyanobacterium *Fischerella* sp. CENA 19 J Appl Phycol 2004 6 237 243

[B76] Raveh A Carmeli S Antimicrobial Ambiguines from the *Cyanobacterium Fischerella* sp. Collected in Israel J Nat Prod 2007 6 196 201 17315959 10.1021/np060495r

[B77] Menéndez JC Chemistry of the Welwitindolinones Top Heterocycl Chem 2007 6 63 101

[B78] Ianora A Bentley MG Caldwell GS Casotti R Cembella AD Engström-Öst J Halsband C Sonnenschein E Legrand C Llewellyn CA Paldavičienë A Pilkaityte R Pohnert G Razinkovas A Romano G Tillmann U Vaiciute D The relevance of marine chemical ecology to plankton and ecosystem function: an emerging field Mar Drugs 2011 6 1625 1648 22131962 10.3390/md9091625 PMC3225939

[B79] Jaki B Orjala J Heilmann J Linden A Vogler B Sticher O Novel extracellular diterpenoids with biological activity from the cyanobacterium *Nostoc commune* J Nat Prod 2000 6 339 343 10757714 10.1021/np9903090

[B80] Ribalet F Berges JA Ianora A Casotti R Growth inhibition of cultured marine phytoplankton by algal-derived polyunsaturated aldehydes Aquat Toxicol 2007 6 219 227 17942163 10.1016/j.aquatox.2007.09.006

[B81] Vardi A Formiggini F Casotti R de Martino A Ribalet F Miralro A Bowler C A stress surveillance system based on calcium and nitric oxide in marine diatoms PLoS Biol 2006 6 411 419 10.1371/journal.pbio.0040060 PMC1370914 16475869

[B82] Balestra C Alonso-Sáez L Gasol JM Casotti R Group-specific effects on coastal bacterioplankton of polyunsaturated aldehydes produced by diatoms Aquat Microb Ecol 2011 6 123 131

[B83] Vedediktov PS Krivoshejeva AA The mechanism of fatty-acid inhibition of electron transport in chloroplasts Planta 1983 6 411 414 10.1007/BF00392076 24258293

[B84] Ikawa M Algal polyunsaturated fatty acids and effects on plankton ecology and other organisms. UNH Center for Freshwater Biol Res 2004 6 17 44

[B85] Cantillo-Ciau Z Moo-Puc R Quijano L Freile-Pelegrin Y The tropica brown alga *Lobophora variegata*: a source of antiprotozoal compounds Mar Drugs 2010 6 1291 1304 10.3390/md8041292 PMC2866487 20479979

[B86] Mizushina Y Kasai N Iijima H Sugawara F Yoshida H Sakaguchi K Sulfoquinovosylacylglycerol, a eukaryotic DNA polymerase inhibito and anti-cancer agent Curr Med Chem Anticancer Agents 2005 6 613 625 16305483 10.2174/156801105774574685

[B87] Santana CM Ferrera ZS Padrón MET Rodríguez JJS Methodologies for the extraction of phenolic compounds from environmental samples: New Approaches Molecules 2009 6 298 320 10.3390/molecules14010298 PMC6253767 19136918

[B88] D’Abrosca B DellaGreca M Fiorentino A Isidori M Monaco P Pacifico S Chemical constituents of the aquatic plant *Schoenoplectus lacustris*: evaluation of phytotoxic effects on the green alga *Selenastrum capricornutum* J Chem Ecol 2006 6 81 96 16525872 10.1007/s10886-006-9354-y

[B89] Nakai S Inoue Y Hosomi M Murakami A *Myriophyllum spicatum*-released allelopathic polyphenols inhibiting growth of blue-green algae Microcystis aeruginosa Water Res 2000 6 3026 3032

[B90] Leão PN Pereira AR Liu WT Ng J Pevzner PA Dorrestein PC König GM Vasconcelos VM Gerwick WH Synergistic allelochemicals from a freshwater cyanobacterium Proc Natl Acad Sci 2010 6 25 11183 11188 20534563 10.1073/pnas.0914343107 PMC2895120

[B91] Mason CP Edwards KR Carlson RE Pgnatello J Gleason RK Wood JM Isolation of chlorine-containing antibiotic from the freshwater Cyanobacterium *Scytonema hofmanni* Science 1982 6 4531 400 402 6800032 10.1126/science.6800032

[B92] Berry JP Gantar M Perez MH Berry G Noriega FG Cyanobacterial toxins as allelochemicals with potential applications asalgaecides, herbicides and insecticides Mar Drugs 2008 6 117 146 18728763 10.3390/md20080007 PMC2525484

[B93] Gross EM Allelopathy of aquatic autotrophs Crit Rev Plant Sci 2003 6 313 339

[B94] Jüttner F Wu JT Evidence of allelochemical activity in subtropical cyanobacterial biofilms of Taiwan Archiv für Hydrobiologie 2000 6 505 517

[B95] Haslarn E Plant Polyphenols. vegetable tannins Revisited 1989 Cambridge: Cambridge University Press

[B96] Winder JS Canneli RJP Walker JM Delbarre S Francisco C Farmer PB Glycosidase inhibitors from algae Biochem. Soc 1989 6 17 1030 1031 10.1042/bst0171030 2516818

[B97] Ilori OJ Ilori OO Allelochemicals:types, activities and usage in Pest control J Sci Sci Ed, Ondo 2012 6 1 106 110

[B98] Hagmann L Jiittner F Fischerellin A a Novel Photosystem-ll-inhibiting Allelochemical of the Cyanobacterium *Fischerella muscicola* with antifungal and herbicidal activity Tetrahedron Lett 1996 6 36 6539 6542

[B99] Entzeroth M Mead DJ Patterson GML Moore RE A herbicidal fatty acid produced by *Lyngbya aestuarii* Phytochemistry 1985 6 2875 2876

[B100] Mitrovic SM Pflugmacher S James KJ Furey A Anatoxin-aelicits an increase in peroxidase and glutathione-S-transferase activity in aquatic plants Aquat Toxicol 2004 6 185 192 15145228 10.1016/j.aquatox.2004.03.017

[B101] Remmel EJ Hambright KD Toxin-assisted micropredation: experimental evidence showsthat contact micropredation rather than exotoxicity is the role of Prymnesium toxins Ecol Lett 2012 6 126 132 22132867 10.1111/j.1461-0248.2011.01718.x

[B102] Raposo MF de Morais RM Bernardo de Morais AM Bioactivity and applications of sulphated polysaccharidesfrom marine microalgae Mar Drugs 2013 6 1 233 252 23344113 10.3390/md11010233 PMC3564169

[B103] Bhadury P Wright PC Exploitation of marine algae: biogenic compounds for potential antifouling application Planta 2004 6 561 578 15221382 10.1007/s00425-004-1307-5

[B104] Hoffman Y Aflalo C Zarka A Gutman J James TY Boussiba S Isolation and characterization of a novel chytrid species (phylum Blastocladiomycota), parasitic on the green alga *Haematococcu*s Mycol Res 2008 6 1 70 81 18222678 10.1016/j.mycres.2007.09.002

